# Interpreting the Mechanism of APOE (p.Leu167del) Mutation in the Incidence of Familial Hypercholesterolemia; An In-silico Approach

**DOI:** 10.2174/1874192401711010084

**Published:** 2017-09-14

**Authors:** Omran Mohammed Rashidi, Fatima Amanullah H.Nazar, Mohamed Nabil Alama, Zuhier Ahmed Awan

**Affiliations:** 1 Department of Clinical Biochemistry. Faculty of Medicine, King Abdulaziz University, Jeddah, Saudi Arabia; 2 Department of Biology, Genomic and Biotechnology Section. Faculty of Science, King Abdulaziz University, Jeddah, Saudi Arabia; 3 Adult interventional cardiology, Cardiology unit, King Abdulaziz University Hospital (KAUH), Jeddah, Saudi Arabia

**Keywords:** Apolipoprotein E (APOE), P. Leu167del, Familial Hypercholesterolemia (FH), Homology modeling, Structural deviation, Molecular Docking Simulation

## Abstract

**Background:**

Apolipoprotein E (APOE) gene is a ligand protein in humans which mediates the metabolism of cholesterol by binding to the low-density lipoprotein receptor (LDLR). P.Leu167del mutation in APOE gene was recently connected with Familial Hypercholesterolemia, a condition associated with premature cardiovascular disease. The consequences of this mutation on the protein structure and its receptor binding capacity remain largely unknown.

**Objective:**

The current study aims to further decipher the underlying mechanism of this mutation using advanced software-based algorithms. The consequences of disrupting the leucine zipper by this mutation was studied at the structural and functional level of the APOE protein.

**Methods:**

3D protein modeling for both APOE and LDLR (wild types), along with APOE (p.Leu167del) mutant type were generated using homology modeling template-based alignment. Structural deviation analysis was performed to evaluate the spatial orientation and the stability of the mutant APOE structure. Molecular docking analysis simulating APOE-LDLR protein interaction was carried out, in order to evaluate the impact of the mutation on the binding affinity.

**Result:**

Structural deviation analysis for APOE mutated model showed low degree of deviance scoring root-mean-square deviation, (RMSD) = 0.322 Å. Whereas Docking simulation revealed an enhanced molecular interaction towards the LDLR with an estimation of +171.03 kJ/mol difference in binding free energy.

**Conclusion:**

This *in-silico* study suggests that p.Leu167del is causing the protein APOE to associate strongly with its receptor, LDLR. This gain-of-function is likely hindering the ability of LDLR to be effectively recycled back to the surface of the hepatocytes to clear cholesterol from the circulation therefore leading to FH.

## 1 INTRODUCTION

Familial Hypercholesterolemia (FH) is a genetic lipoprotein disorder characterized by elevated serum low-density lipoprotein cholesterol (LDL-C). It is inherited either as an autosomal dominant (AD) or as an autosomal recessive (AR) hypercholesterolemia. The worldwide prevalence of the heterozygous form is 1 in 500, while the homozygous is 1 in 1,000,000 [1]. In special populations there is an evidence that it is even more common due to inbreeding (French-Canadian) and consanguinity (Lebanese) [1]. Excessive levels of LDL-C can increase the risk of premature coronary heart disease through acceleration of atherosclerosis [2]. Genes associated with AD-FH phenotype involve: the low density lipoprotein receptor (*LDLR*) [2, 3], apolipoprotein B (*APOB*) [4] and proprotein convertase subtilisin/kexin type 9 (*PCSK9*) [5]. The AR form of FH was found to be caused by the very rare loss-of-function mutations in the low-density lipoprotein receptor adapter protein 1 (*LDLRAP1)*gene [6].

Genome-wide association studies (GWAS) have reported that the Apolipoprotein E (*APOE*) gene as strongly associated with LDL-C levels [7]. APOE is a multifunctional plasma protein that is regularly synthesized in several organs such as the liver, brain, kidney and spleen [8]. It is an essential component that mediates binding of all lipoproteins [9, 10]. The APOE-LDLR protein complex contributes significantly to cholesterol levels in the regular cycle of lipid metabolism [11]. The *APOE* gene exists in 3 major allelic variants; E2, E3 and E4, where E3 is considered to be the normal form, while other alleles have been associated with various diseases.

The p.Leu167del mutation was first reported by Marduel *et al.* to be strongly associated with a large family of positive autosomal dominant hypercholesterolemia (ADH) occurrence [12]. Later on, Awan **et al.** presented how p.Leu167del structurally altered the LDLR binding domain within the APOE protein. This mutation disrupted the leucine zipper near its 4th α-helical structure, which is structurally kown to be highly critical region in the protein. The previous observation was registered *via* computational biology using established template-based protein modeling, followed by 3D structure alignment of both the reference and Leu167del proteins [13].

The purpose of the current study is to decipher the underlying mechanism of p.Leu167del mutation using advanced software-based algorithms. We are aiming to further elucidate the consequences of disrupting the leucine zipper at the structural and functional levels of the APOE protein.

## 2 MATERIALS AND METHODS

### 2.1 Data Set

The reference genomic data for human *APOE* and *LDLR* genes were obtained from the National Center for Biological Information (NCBI). Corresponding amino acid sequences of APOE protein (CCDS Transcript ID: ENST00000252486.8) and LDLR protein (CCDS Transcript ID: ENST00000558518.5) were retrieved from Ensemble genome browser (http://www.ensembl.org/index.html**)**.

### 2.2 3D Protein Modeling

Crystalized protein structures of both candidate genes were absent from all protein and allied genomic databases. Therefore, computational modeling based on homology prediction was utilized to construct the reference protein structures. I-Tasser web server (http://zhanglab.ccmb.med.umich.edu/I-TASSER/**)** was employed in the modeling procedure. Protein models were generated based on multiple sequence alignment and fragment assembly simulations. I-Tasser generated 5 protein data bank (PDB) formatted models for each sequence. Only the top model with the highest confidence score (C-score), accompanied with the most reliable measurement of template modeling score (TM-scores) were selected for the follow-up analysis.

Predicted reference models of APOE and LDLR were subsequently subjected to energy minimization using DeepView-Swiss PDB Viewer (http://spdbv.vital-it.ch/**)**. This software depends on GROningen MOlecular Simulation (GROMOS) 96 force field techniques to release local constraints at their local residues [14]. It is also noteworthy that the p.Leu167del mutation was manually curated in the referenced protein sequence of APOE before it was submitted for automodeling using Swiss model: automated homology modeling (https://swissmodel.expasy.org/) [15].

### 2.3 Protein Structural Divergence Analysis

The level of structural deviance of the mutant APOE protein was homologically established using protein structural alignment in accordance with the wild-type model. Using Pymol molecular viewer****(**https://www.pymol.org/**)**, sequence alignment was performed followed by a structural superposition of the mutant and wild-type PDB files. The bonds of alpha carbon (Cα) atoms of the backbone peptide are more often considered for their structural alignment. They usually experience minimal variant confirmations. Pymol proceeded in multiple cycles of refinement and structural screening, ending with the calculation of Root Mean Square Deviation (RMSD). This score can illustrate the structural drift to the polypeptide chain level when both mutant and the wild type models are structurally aligned. Structural deformities induced by genetic mutations as p.Leu167del are usually affecting RMSD scores, and therefore model structures [16].

### 2.4 Molecular Docking Simulation

The Hex dock server (http://hex.loria.fr/) was used as an interactive molecular program. This server is capable of calculating docking models of complex protein pairs from their PDB format [17]. Predicted APOE and LDLR protein models were both submitted to the server into 2 separate protein complex sets; wild (LDLR + wild-type APOE) and mutant (LDLR + mutated APOE). Initially, polar hydrogen atoms were turned off, while the intermolecular axis was activated in order to identify the centroids of the protein models. As Hex reads the PDB structure, it uses its all-atom centre of mass as its centroid. These centroids are used as the local coordinate origin for docking [17].

Ellipsoid axes were assigned to recognise the difference in structural parameters, especially when protein complexes are in binding mode. Hex performed a 6-dimensional search over the full rotational ranges around each protein complex. Energy calculations were set at 180°, allowing for total rotational increments for both receptor (LDLR) and ligand (APOE) around their own centroids. The default grid spacing was set at a high-resolution scoring of 0.5 Å. The steric scan (n=16) phase of the docking was calculated to proceed at (1+40)/0.75=53 intermolecular separations, in +/- steps of 0.75 Å. The highest scoring scan orientations were applied to the final search phase (n=25) and the final energies were calculated in Kilojoules units. In the light of the above, any abnormalities regarding the binding affinity of mutant APOE protein model will be distinguished.

## 3 RESULTS

### 3.1 Protein Models

I-Tasser server predicted the models of complete peptide chains of APOE wild and LDLR *via* homological protein structures. The criteria for selecting the best models for further analysis depends on the confidence C-scores that typically ranges from -5 to 2. In addition to the highest TM-score, which usually ranges from 0 to 1. The score >0.5 resembles a model featuring similar structural characteristics to its template-based model.

**Fig. (1):**
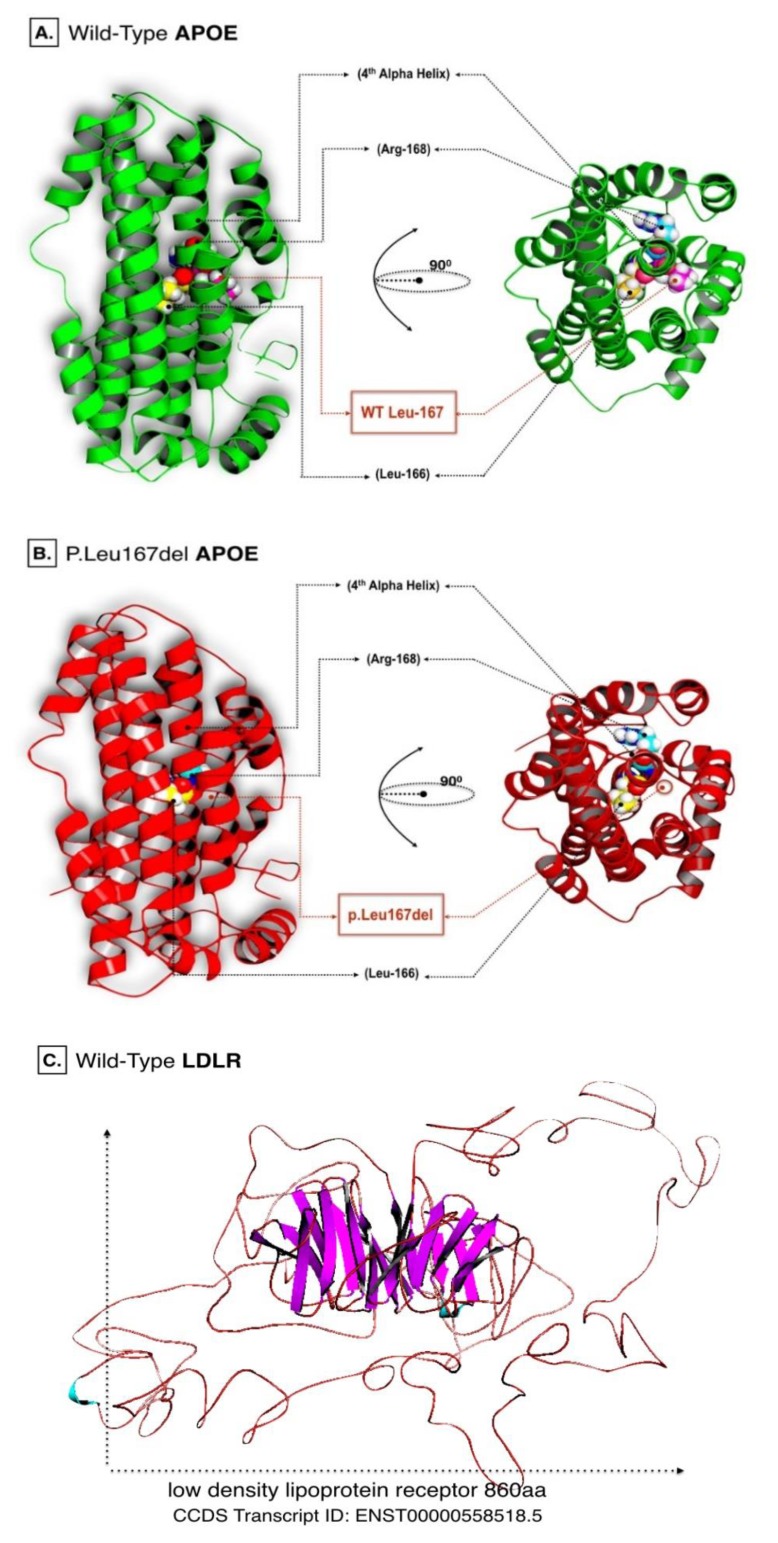
(A). Protein 3D model of wild type APOE with emphasis on the Leu167aa position (B). Protein 3D model of the APOE p.Leu167del with emphasis on Leu167aa deletion from the model (C). Protein 3D model of the LDLR.

C-score predicted by I-Tasser were equal to -0.50 and 0.04 for wild APOE and LDLR models respectively. However, the estimated TM-score for wild APOE resulted in 0.65±0.13, while 0.72±0.11 for the LDLR model. Energy minimization helped to repair distorted geometries and eliminate bad contacts in targeted proteins. Such analysis is usually recommended prior to performing computational docking simulations. Energy minimized models of APOE wild and LDLR are shown in (Fig. **1**).

The mutated file of APOE model was generated instantly and by automation using wild type PDB file of wild APOE.

### 3.2 Structural Deviation Analysis

**Fig. (2):**
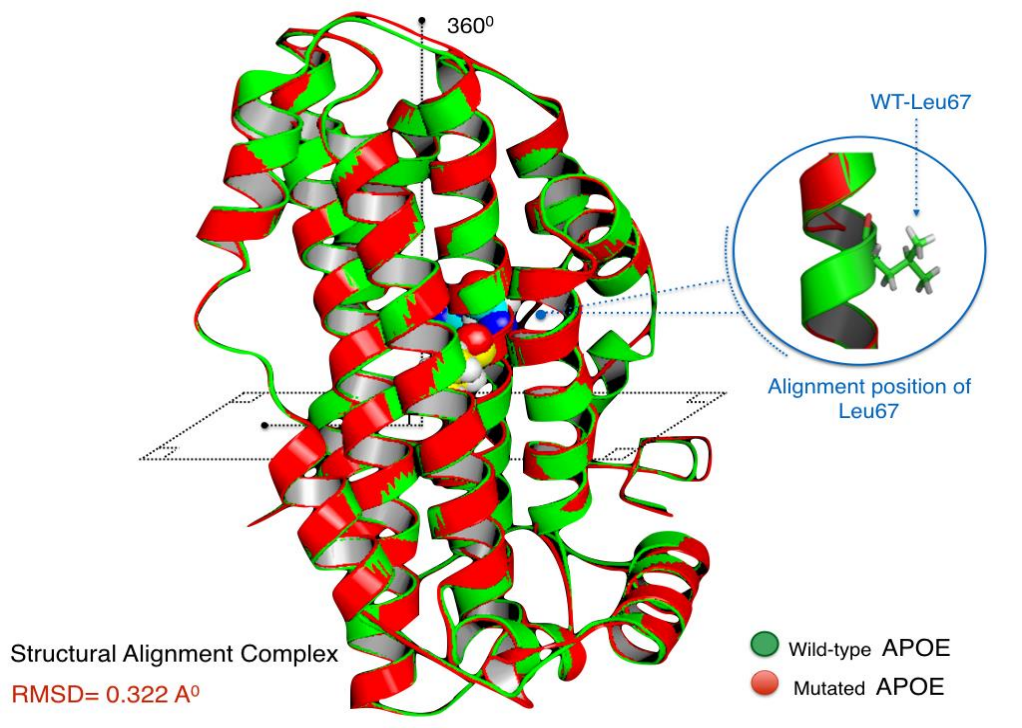
APOE wild and mutated form are in a superimposed position.

Aligned APOE models (wild/mutant) have yielded RMSD scores. Normally, RMSD is estimated to be <2.0 Å in the deviation analysis of protein structure. Computed RMSD values represent the average distance between the Cα backbone atoms (Fig. **2**). A considerable degree of structural deviance with RMSD equalling to 0.322 Å was obtained from this analysis.

### 3.3 Docking Analysis

Docking the wild type APOE model revealed an enhanced binding activity toward its receptor; LDLR, with an estimated binding energy of -643.33 kJ/mol. However, docking simulation of the mutated APOE model to the LDLR revealed an estimation binding energy of -472.30 kJ/mol. Interestingly, the docking simulation has revealed that the mutated APOE model is showing an increased molecular interaction towards the LDLR receptor with an estimation of +171.03 kJ/mol difference in binding free energy. Hex binding calculation results for both protein complexes are shown in (Table **1**).

## 4 DISCUSSION

The field of structural analysis has significantly evolved in the quest to propose a systematic and cost-effective answer to some ambiguous issues related to biological systems. The consequence of some genetic mutations on these systems is one of the main issues that have been addressed [18]. Nowadays, the computational pipeline is widely supported with software-based algorithms. Protein-protein interaction simulation programs can confidently predict functional abrogation.

Regarding FH, computational biology has proven to be effective to illustrate a plausible variation in the genome which might indicate some pathogenicity to cause the disease phenotype [19, 20] [21, 22]. APOE protein, in particular, has been the subject of structural analysis in multiple studies [23, 24]. Awhile after the GWAS report of a locus in *APOE* gene to be highly associated with ADH, several separate studies investigated cases and families with the similar condition [12, 13, 25]. However, p.Leu167del mutation did not undergo extensive computational interpretation. The impact of this mutation on the structure and function of the protein can go through further investigation using advanced *in-silico* software specifically trained to test the effects of genetic mutations at the protein level. The current study provides additional insights on p.Leu167del mutation which has been reported to cause ADH and FH. Following our previous work [13], we adopted herein an approach, alternative to the traditional *in-vivo* and *in-vitro* studies, to elucidate the consequences of p.Leu167del mutation in APOE protein attributes at the molecular and cellular levels.

Although in-frame genetic mutations (*small indels or insertions and deletions*) do not alter the sequence of amino acid in the protein chain, Human Gene Mutation Database (HGMD Professional release 2015.4) reported >4000 disease-causing in-frame indels, corresponding to 2.2% of all mutations. The 1000 Genomes Project Consortium reported that 1.4% of detected exonic variants were indels [26]. Notably, functional and population annotations for these in-frame indels are becoming increasingly available [27]. Our current research also revealed that in-frame p.Leu167del has a significant, possibly pathogenic impact on its protein.

In a former study [13], we have initially assessed the impact of this deletion on the 3D structure of APOE using online protein function prediction tools (SIFT and PolyPhen). The study hypothesized that the changed leucine zippers motif might weaken the lipoprotein particle, and prevent it from binding properly with its receptor, LDLR [13]. Leucine zippers are common helical motifs with periodic segments containing leucine residues in protein compounds [28]. Mainly they are featured in eukaryotic transcription factors as they contain highly conservative regions for DNA-protein binding activity [29]. However, they are also being annotated as one of the critical protein modules for protein-protein interactions [30]. Many tools such as SIFT and PolyPhen can predict the likely pathogenicity of certain mutations. However, the low specificity of these programmes undermines the level of their accuracy prediction [31].

Structural deviation analysis is often utilised to properly evaluate the spatial orientation of the mutant proteins, where certain degrees of residual deviance can compromise the stability and the dynamical state of protein structures, which in turn, can affect their biological function [32]. Herein, the structural deformity induced by this mutation, although disrupting the leucine zipper at the 4th α-helix, had a very minimal effect on the protein. RMSD is only a quantitative measurement of the degree of similarity of protein structures [33]. The threshold of structural deviance is usually considered as <2.0 Å [34]. Therefore, p.Leu167del does not seem to impact the APOE at a critical level.

The presence of deformed abnormalities can also affect the binding affinity of targeted protein when interacting with partner molecules [35]. Therefore, computational molecular docking has been a highly desirable method. It is specifically aimed to predict a 3-dimensional simulation of complex protein-protein interaction [36]. As shown above, this approach can simulate the actual ligand-receptor interaction to predict calculated energies that define the strength of their association [37].

The increased difference, around +171 kJ/mol in the binding energy between the mutated form of the APOE protein and LDLR was remarkable Fig. (**3**). While most deletion mutations altering proteins binding affinity may cause significant disturbance to the function of the protein, p.Leu167del on the other hand was interestingly found to influence APOE oppositely. The detected protein is strongly attached to its receptor protein; LDLR. Note that the LDLR protein is the only member of the LDL receptor superfamily to demonstrate high affinity for binding with the wild type APOE protein [38]. This makes our mutation of interest to be classified as a gain-of-function (GOF) mutation.

The interaction activity of the APOE-LDLR complex has been pointed as an essential cellular activity for mediation of cholesterol metabolism, as the functional interaction between both proteins had already been associated with the risk of CVD [39].

The results of the present study are consistent with a recently published report [40]. Cenarro *et al.* proposed that the association of p.Leu167del with ADH appears to be due to the down-regulation of the LDL receptor [40]. This can indicate that p.Leu167del resembles the characteristics of a GOF mutation in APOE protein. Similar to APOE (E4) isoform, that also negatively modulate the recycling capacity of the LDLR preventing cholesterol clearance from the circulation [41].

Eventually, the impact of p.Leu167del mutation on the binding activity of APOE might initiate a quest for investigating a possible therapeutic target. Further research is needed to better understand *APOE* gene normal and variant-related biological aspects leading to FH.

## CONCLUSION

The mechanism of p.Leu167del in regards to hypercholesterolemia was demonstrated here by this advance computational analysis. The p.Leu167del mutation was shown to exert an increased force of attraction, which triggers the mutated APOE protein to strongly associate with the LDLR protein. This can hinder the ability of the LDLR to be effectively recycled back to the surface of hepatic cells. Subsequently, a decrease in LDL endocytosis results in an increase in LDL particles circulating in the plasma. These high cholesterol levels will increase the risk of atherosclerosis.

## Figures and Tables

**Fig. (3):**
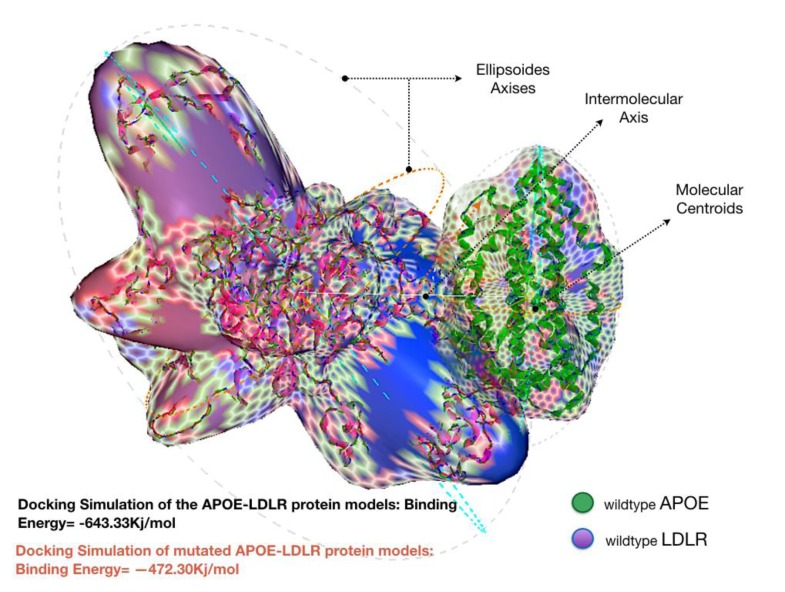
An illustration of the wild-type protein complex of APOE-LDLR docking simulation.

**Table 1:** Calculated binding energy of both wild-type and mutated protein complexes as predicted by the hex doc server.

**Interacting Protein Complex**		**Total Docking Energy (kJ/mol)**	**Differences in Docking Energy**	**Level of Binding Affinity**
**Receptor Protein**	**Ligand Protein**
LDLR	APOE wild type	-643.33	**-**	**-**
	APOE p.Leu167del	-472.3	**171.03**	**↑↑**
